# Thrombomodulin facilitates peripheral nerve regeneration through regulating M1/M2 switching

**DOI:** 10.1186/s12974-020-01897-z

**Published:** 2020-08-21

**Authors:** Tzu-Chieh Huang, Hua-Lin Wu, Szu-Han Chen, Yun-Ting Wang, Chia-Ching Wu

**Affiliations:** 1 Department of Cell Biology and Anatomy, College of Medicine, National Cheng Kung University, No. 1, University Rd, Tainan, 701 Taiwan; 2 Institute of Basic Medical Sciences, College of Medicine, National Cheng Kung University, Tainan, Taiwan; 3 International Center for Wound Repair and Regeneration, National Cheng Kung University, Tainan, Taiwan; 4 Department of Biochemistry and Molecular Biology, College of Medicine, National Cheng Kung University, Tainan, Taiwan; 5 Division of Plastic and Reconstructive Surgery, Department of Surgery, National Cheng Kung University Hospital, College of Medicine, National Cheng Kung University, Tainan, Taiwan; 6 Institute of Clinical Medicine, College of Medicine, National Cheng Kung University, Tainan, Taiwan; 7 Department of Biomedical Engineering, National Cheng Kung University, Tainan, Taiwan

**Keywords:** Thrombomodulin, Macrophage polarization, Peripheral nerve regeneration, Inflammation, IL-4 receptor, STAT6

## Abstract

**Background:**

Excessive inflammation within damaged tissue usually leads to delayed or insufficient regeneration, and nerves in the peripheral nervous system (PNS) generally do not recover fully following damage. Consequently, there is growing interest in whether modulation of the inflammatory response could help to promote nerve regeneration in the PNS. However, to date, there are no practical therapeutic strategies for manipulating inflammation after nerve injury. Thrombomodulin (TM) is a transmembrane glycoprotein containing five domains. The lectin-like domain of TM has the ability to suppress the inflammatory response. However, whether TM can modulate inflammation in the PNS during nerve regeneration has yet to be elucidated.

**Methods:**

We investigated the role of TM in switching proinflammatory type 1 macrophages (M1) to anti-inflammatory type 2 macrophages (M2) in a human monocytic cell line (THP-1) and evaluated the therapeutic application of TM in transected sciatic nerve injury in rats.

**Results:**

The administration of TM during M1 induction significantly reduced the expression levels of inflammatory cytokines, including *TNF-a* (*p* < 0.05), *IL-6* (*p* < 0.05), and *CD86* (*p* < 0.05), in THP-1 cells. Simultaneously, the expression levels of M2 markers, including *IL-10* (*p* < 0.05) and *CD206* (*p* < 0.05), were significantly increased in TM-treated THP-1 cells. Inhibition of IL-4R-c-Myc-pSTAT6-PPARγ signaling abolished the expression levels of *IL-10* (*p* < 0.05) and *CD206* (*p* < 0.05). The conditioned medium (CM) collected from M1 cells triggered an inflammatory response in primary Schwann cells, while CM collected from M1 cells treated with TM resulted in a dose-dependent reduction in inflammation. TM treatment led to better nerve regeneration when tested 6 weeks after injury and preserved effector muscle function. In addition, TM treatment reduced macrophage infiltration at the site of injury and led to potent M1 to M2 transition, thus indicating the anti-inflammatory capacity of TM.

**Conclusions:**

Collectively, our findings demonstrate the anti-inflammatory role of TM during nerve regeneration. Therefore, TM represents a potential drug for the promotion and modulation of functional recovery in peripheral nerves that acts by regulating the M1/M2 ratio.

## Background

Although the peripheral nervous system (PNS) exhibits superior regenerative capacity compared to the central nervous system (CNS), the regeneration of peripheral nerves is usually slow and often incomplete. Inflammation has been identified as a therapeutic target for peripheral nerve regeneration; by regulating inflammation, it may be possible to improve peripheral nerve repair. During the inflammatory phase, the synthesis of proinflammatory cytokines, such as tumor necrosis factor-α (TNFα), interleukin-6 (IL-6), and IL-1β, increases significantly [1]. The increased levels of inflammatory cytokines then induce the recruitment of phagocytes to remove myelin debris. A previous study showed that mice lacking Toll-like receptor 2 (TLR2) or TLR4 failed to produce IL-1β following nerve ligation. Furthermore, these mice showed reduced levels of macrophage infiltration at the injury site, thus resulting in the impairment of myelin clearance, axonal regeneration, and functional recovery [2]. Other research showed that the elimination of CD11b-positive cells in injured nerves abrogated the immune response, thus resulting in a poorer outcome [3]. However, injury-induced inflammation may develop into chronic inflammation and cause undesired symptoms, including neuropathic pain [4]. As a result of the double-edged properties of inflammation, there is growing interest in targeting the modulation of inflammation for the treatment of nerve injuries [5].

Thrombomodulin (TM) is a transmembrane glycoprotein containing five domains: a lectin-like domain, an endothelial growth factor (EGF)-like domain, a serine-threonine-rich domain, a transmembrane domain, and a cytoplasmic tail. Early evidence supporting the potential involvement of TM in the suppression of tissue inflammation was the finding that the endocytosis of TM at the site of injury led to inflammation [6]. Subsequent studies have provided further direct evidence of the anti-inflammatory properties of TM. TM is believed to suppress inflammation via a protein C (PC)-dependent pathway. When binding to the EGF-like domain of TM, the ability of thrombin to activate the PC pathway is amplified by more than 1000-fold [7]. Activated PC (APC) then exhibits a wide range of anti-inflammatory activities, including the reduction in neutrophil infiltration and the downregulation of proinflammatory cytokines [8]. APC is also known to protect the endothelial cell barrier and increase the expression of anti-inflammatory cytokines [8]. Nevertheless, there is evidence showing that the generation of APC is not the only mechanism underlying the anti-inflammatory activity of TM. A previous article demonstrated that mice with mutant TM that failed to generate APC exhibited a similar pulmonary response to LPS compared to wild-type mice [9]. This finding reveals that TM can suppress inflammation in an APC-independent mechanism. Interleukin-4 receptor (IL-4R) and its downstream signal transducer and activator of transcription 6 (STAT6) are critical for inflammation regulation [10]; however, the interaction between TM and IL-4R-mediated anti-inflammation has yet to be studied. Furthermore, the lectin-like domain of TM is known to exert an anti-inflammatory function by interfering with the adhesion of neutrophils to endothelial cells by suppressing the activation of extracellular signal-regulated kinase (ERK) 1/2 [11]. TM can also impede the lipopolysaccharide (LPS)-induced M1-like macrophage phenotype by interrupting the binding between LPS and CD14 [12]. However, the precise function of TM in promoting M1/M2 switching has yet to be elucidated.

Macrophages carry out a wide range of functions during both nerve degeneration and regeneration, including the removal of cell debris [13], the regulation of Schwann cell (SC) migration [14], the enhancement of axon elongation [15], and the promotion of remyelination [16]. A previous study demonstrated that the successful activation of macrophages is a prerequisite for efficient nerve repair [17]. Based on their properties, macrophages can be generally categorized into two phenotypes: proinflammatory M1 macrophages and proregenerative M2 macrophages. Both of these macrophage phenotypes are required during nerve repair. A previous study reported the disturbance of M1 polarization following nerve crush injury. This was due to the inhibition of CD300f, an immunoreceptor that is expressed at high levels by M1 macrophages; this led to a reduction in axon regeneration and delayed functional motor recovery [18]. Similarly, depletion of CD206^+^ M2 macrophages is known to prolong inflammation within injured tissue [19]. Although macrophages with different phenotypes are required for successful nerve regeneration, a prolonged period of M1 activation has been shown to restrict nerve growth [20]. Previous studies have shown that bridging a transected nerve with a scaffold containing interferon-γ (IFN-γ) can reduce the infiltration of SCs at the site of transection [20]. In contrast, the use of a nerve scaffold that can favor M2 polarization has been shown to improve SC infiltration and enhance the growth of axons [21]. According to previous studies, regulating inflammation might be a better option for functional nerve repair than total depletion. However, this would require the development of specific immunomodulatory factors. Currently, there are several preclinical and clinical drugs that target macrophages and promote M2 polarization. However, many of these drugs were designed to treat autoimmune diseases, such as rheumatoid arthritis and inflammatory bowel disease [22]. TM is now undergoing phase III clinical trials in the USA, the European Union, Asia, and other regions [23]. Moreover, recombinant TM is known to exhibit a neuroprotective effect created by the inhibition of apoptosis and the reduction in reactive oxygen species (ROS) [24, 25]. It has also been demonstrated that recombinant TM ameliorates neuropathic pain [26], thus making recombinant TM a potential immunoregulator for the regeneration of nerve tissue.

Identifying a means of immunomodulating the M1/M2 switch is critical for the development of therapeutic strategies to regulate the nerve regeneration process. In the current study, we investigated the effect of TM treatment on the transition between M1 and M2 macrophages. We also evaluated the therapeutic outcome of TM treatment with regard to nerve repair and functional remyelination following peripheral nerve injury. Finally, we investigated the mechanisms underlying these effects and the beneficial roles of TM-induced M2 macrophages on peripheral nerve regeneration.

## Materials and methods

### THP-1 cell maintenance and differentiation

Human monocytic cell line (THP-1) was used to test the transition of macrophage polarization following TM application. THP-1 cells were obtained from the Bioresource Collection and Research Center (BCRC, Taiwan). The cells were maintained in Roswell Park Memorial Institute (RPMI)-1640 medium (Himedia, USA) containing 10% fetal bovine serum (FBS) (HyClone, USA) and 1% penicillin streptomycin (Gibco, USA). M1 macrophages were induced in accordance with a previously described protocol, with minor modifications [27]. THP-1 cells, at a seeding density of 1.5 × 10^5^ cells/ml, were incubated with RPMI-1640 medium containing 150 nM phorbol 12-myristate 13-acetate (PMA) (Sigma, USA) for 24 h, followed by incubation in PMA-free RPMI-1640 medium for an additional 24 h to reach the M0 stage. Subsequently, the M0 macrophages were induced to progress toward the M1 phenotype by incubation in RPMI-1640 medium containing 20 ng/ml IFN-γ (R&D system, USA) and 100 ng/ml lipopolysaccharide (LPS, Sigma, USA) for 1 day.

### In vitro macrophage polarization and the application of TM

**Fig. 1:**
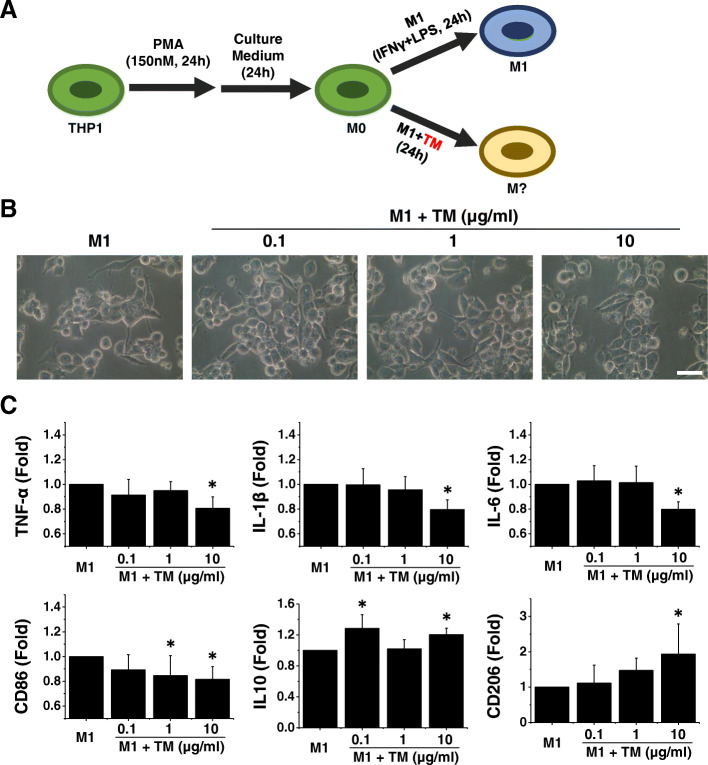
Thrombomodulin (TM) enhances M2 macrophage polarization in the presence of inflammatory cytokines. **a** A schematic diagram of the THP-1 induction protocol demonstrating the process of macrophage differentiation. **b** Representative light microscopy images revealed that TM-treated M1 macrophages exhibited no apparent change in morphology compared with M1 macrophages. Scale bar, 10 μm. **c** The expression levels of M1 and M2 markers were tested by using quantitative RT-PCR. The quantitative RT-PCR data demonstrated that the addition of TM to the M1 induction medium disrupted M1 polarization and enhanced polarization toward the M2 phenotype. *n* = 5. Mean ± SD. *p < 0.05 compared with M1

M1 macrophages were induced by using M1 induction medium, with or without TM (Fig. 1a). Recombinant TM, TM domain 1 (TMD1), and TM domain 23 (TMD23) were prepared as published previously [12] and were kindly provided by Dr. Hua-Lin Wu (National Cheng Kung University, Taiwan). According to previous literature, the administration of TM (2, 10, and 50 μg/ml) can block LPS-induced platelet-dependent neutrophil extracellular trap formation; notably, a concentration of only 2 μg/ml of TM was able to induce this effect [28]. Based on these previous findings, we used 0.1, 1, and 10 μg/ml of TM in the present study. For TMD1 and TMD23, we used 300 nM of each in the present study. Then, 24 h after TM treatment, we extracted both RNA and protein from cells to define the phenotype of the macrophages.

### Primary SC isolation and inflammatory responses

SCs were isolated as described previously [29]. First, we isolated sciatic nerves from eight 6-week-old Sprague-Dawley (SD) rats. To avoid contamination by fibroblasts, we removed the epineurium from the nerves using fine forceps. Next, we teased the nerve until individual fibers were evident. The teased nerves were then digested overnight at 37 °C with 0.25% Dispase II (Invitrogen, USA) and 0.05% type I collagenase (Invitrogen, USA). To remove myelin debris, the digested fibers were first mechanically torn into small fragments and then seeded onto dishes as drops. Finally, isolated SCs were maintained in Dulbecco’s modified Eagle’s medium (DMEM) high glucose medium (HyClone, USA) containing 10% FBS, 10 nM neuregulin (R&D system, USA), and 2 μm forskolin (Sigma, USA).

### Preparation of conditioned medium (CM) derived from differentiated macrophages

Cells at different stages (M0, M1, and TM-treated M1) were induced as described above. Following induction, differentiated macrophages were washed with phosphate-buffered saline (PBS) and subsequently placed in fresh RPMI-1640 media containing 1% FBS. Twenty-four hours later, the conditioned medium was collected and centrifuged at 200×*g* for 5 min.

### Creation of an animal model for sciatic nerve regeneration

Male SD rats weighing 225–250 g were purchased from BioLASCO Taiwan Co., Ltd., and maintained at the National Cheng Kung University (NCKU) Animal Center. Animal care and all experimental procedures were performed in accordance with the guidelines provided by the Institutional Animal Care and Use Committee (IACUC) at NCKU; all experimental procedures were approved by this committee (IACUC approval number: 105224). Seven days after arrival, we randomly assigned rats into 3 groups: a sham group, a PBS group, and a 10 μg/ml TM group. Rats in the PBS and TM groups were subjected to nerve transection and conduit reconnection surgery. Rats were first anesthetized by isoflurane inhalation and then given an intraperitoneal injection of Zoletil (1 ml/kg) (Virbac, France) and Ropum (0.1 ml/kg) (Bayer, Germany). The left sciatic nerve was then exposed at the mid-thigh level. A complete transection injury was then created 1 cm distal to the sciatic notch. Immediately after the injury, we reconnected the nerve ends with a 1.5-cm silicon conduit by suturing the ends 0.15 cm into the conduit, leaving a 1.2 cm gap between the stumps. In the sham group, the left sciatic nerve was exposed without nerve transection. The rats were then maintained for 6 weeks to allow the nerves to regrow. We then harvested the transected nerves and performed histological assessments to evaluate nerve growth. We also isolated gastrocnemius muscle from the cuff area on both sides of the rats to investigate functional recovery in the nerves; these muscles were weighed after isolation. For each rat, the gastrocnemius muscle from the left side was normalized to that on the right side to calculate the relative muscle weight (RMW).

### Measurement of gene expression and luciferase reporter assay

The mRNA expression of target inflammation genes and potential M1/M2 markers during TM treatment was evaluated by quantitative polymerase chain reaction (qPCR). In brief, total RNA was isolated from THP-1 cell-derived macrophages using a standard TRIzol (Invitrogen, USA) extraction method. The quality of the isolated RNA was then determined using a Nabi-UV/Vis Nano Spectrometer (MicroDigital Co., Ltd., Korea). To obtain cDNA, total RNA was then reverse-transcribed using Oligo(dT) primers (Invitrogen, USA), dNTP Mix (Invitrogen, USA), 5× First Strand Buffer (Invitrogen, USA), SuperScript III Reverse Transcriptase (Invitrogen, USA), RNaseOUT Recombinant Ribonuclease Inhibitor (Invitrogen, USA), and DTT (Invitrogen, USA), in accordance with the manufacturer’s protocol (SuperScript III CellsDirect cDNA Synthesis System). The target genes were *TNF-α*, *IL-1β*, *IL-6*, *CD86*, *IL-10*, and *CD206*. The specific sequences of the primers are listed in Table 1.

**Table 1:** The specific sequences of the primers

TNFα	Forward	TCAACCTCCTCTCTGCCATC
	Reverse	CCAAAGTAGACCTGCCCAGA
IL1β	Forward	CTGTCCTGCGTGTTGAAAGA
	Reverse	CTGCTTGAGAGGTGCTGATG
IL6	Forward	AGGAGACTTGCCTGGTGAAA
	Reverse	CAGGGGTGGTTATTGCATCT
CD86	Forward	GACGCGGCTTTTATCTTCAC
	Reverse	CCCTCTCCATTGTGTTGGTT
IL10	Forward	CTGCCTAACATGCTTCGAGA
	Reverse	GCATCACCTCCTCCAGGTAA
CD206	Forward	GATGGGTGTCCGAATCTCAG
	Reverse	TTCCACCTGCTCCATAAACC
GAPDH	Forward	CATCAAGAAGGTGGTGAAGC
	Reverse	TGACAAAGTGGTCGTTGAGG

PPARγ response element (PPRE) activity in TM-treated M1 cells was tested by using a luciferase reporter assay. Differentiated THP-1 cells were transfected with the PPRE luciferase reporter by using Lipofectamine 3000 (L3000-015, Invitrogen, USA) [30]. The activity of the PPRE luciferase reporter was determined by the Luciferase Assay System (E1500, Promega, USA) in accordance with the manufacturer’s protocol.

### Protein expression analysis of inflammatory mediators

THP-1 cells were rinsed twice with ice-cold PBS and lysed with RIPA buffer (150 mM NaCl, 1 mM EGTA, 50 mM Tris pH 7.4, 10% glycerol, 1% Triton X-100, 1% sodium deoxycholate, 0.1% SDS, and protease inhibitor cocktail). The protein concentration of the cell lysates was measured using the Bradford reagent (Thermo Fisher, USA). Next, 30 μg of each protein sample was separated by 10% sodium dodecyl sulfate polyacrylamide gel electrophoresis (SDS-PAGE) and then transferred onto nitrocellulose (NC) paper. The resultant NC papers were then incubated overnight at 4 °C with a range of specific primary antibodies, including signal transducer and activator of transcription (STAT6) (1:1000, ab32520, Abcam, USA), p-STAT6 (1:1000, ab28829, Abcam, USA), peroxisome proliferator-activated receptor γ (PPARγ) (1:1000, #2435, Cell Signaling, USA), high mobility group protein B1 (HMGB1) (1:5000, ab18256, Abcam, USA), and interleukin-4 receptor (IL-4 R) (1:1000, ab203398, Abcam, USA). After incubation with the indicated primary antibody, the proteins of interest were then incubated with a horseradish peroxidase (HRP)-conjugated secondary antibody (Millipore, USA) and visualized by the ECL detection system (Millipore, USA).

### Inhibition of c-Myc, p-STAT6, and PPARγ

TM-treated M1 macrophages were incubated with 60 μM c-Myc inhibitor (Calbiochem EMD Biosciences, USA) to disrupt the c-Myc-Max interaction and thereby inhibit the transactivation of the c-Myc target gene. Ten and 100 nM STAT6 inhibitor AS1517499 (Cayman, USA) were used to inhibit the phosphorylation of STAT6. PPARγ was antagonized by 0.1, 1, and 10 μM PPARγ antagonist GW9662 (Cayman, USA).Twenty-four hours after treatment, RNA was extracted from the cells to define the effect of c-Myc, pSTAT6, and PPARγ on macrophage polarization following TM supplementation.

### Detection of ROS levels

The levels of ROS in TM-treated M1 macrophages were determined with a Cellular ROS Assay Kit (DCFDA/H2DCFDA) (Abcam, USA) in accordance with the manufacturer’s protocol. In brief, 2.5 × 10^5^ THP-1 cells were seeded into each well of a 4-well μ-slides (#80426, ibidi, USA). THP-1 cells were then induced into M1 or TM-treated M1 macrophages, as described earlier. To determine ROS levels, we incubated cells with 25 μM DCFDA for 45 min at 37 °C. After incubation, the levels of ROS were determined by fluorescence using confocal microscopy (IX81, Olympus, Japan).

### Toluidine blue staining and transmission electron microscopy

To investigate the specific structure of regenerating nerves, isolated nerves were prefixed with a solution containing 4% paraformaldehyde (Sigma, USA) and 2.5% glutaraldehyde (Sigma, USA) for at least 12 h. Samples of nerves were then postfixed by immersion in 1% osmium tetroxide for 1 h, followed by a PBS wash for an additional 1 h. To avoid shrinkage of the samples during dehydration, all of the nerves were serially dehydrated in 50, 75, 85, 95, and 100% ethanol, followed by immersion in 100% propylene oxide for 30 min. Dehydrated samples were then immersed in a 1:1 solution of propylene oxide and Epon for 2 h. Next, the samples were embedded in pure Epon and baked overnight at 65 °C. We then prepared semithin (2 μm) sections from each specimen. These sections were attached to glass slides and stained with toluidine blue to demonstrate myelination. Three images were randomly acquired from each section, using a ×40 objective (*n* = 3). We counted all remyelinated axons in each microscopic field analyzed. To provide a more detailed representation of nerve structure, we prepared ultrathin sections (80 nm) and processed them with 2% lead citrate and 1% uranyl acetate to enhance image contrast under transmission electron microscopy (JEM-2100, Japan).

### Immunohistochemistry staining

To determine the specific phenotype of macrophages, tissue samples were fixed with 4% paraformaldehyde for 12 h and dehydrated with a series of ethanol concentrations (50–100%). Samples were cleared five times with xylene (30 min on each occasion) and embedded five times in paraffin at 65 °C (30 min on each occasion). Paraffin-embedded tissues were then kept at room temperature, allowing the specimens to solidify. Then, the paraffin sections were sectioned (12 μm) and rehydrated with xylene and ethanol. Next, the sections were incubated with 0.1% Triton (Thermo Fisher, USA), in order to retrieve antigens, and blocked with 1% horse serum (Thermo Fisher, USA). The sections were then incubated overnight with specific primary antibodies to target specific cell populations of interest, including the neuronal markers (PGP9.5 (1:500, ab109261, Abcam, USA), S100B (1:500, ab52642, Abcam, USA), and glial fibrillary acidic protein (GFAP) (1:500, AB5804, Millipore, USA)), macrophage markers (CD68 (1:500, ab125212, Abcam, USA), CD86 (1:500, ab53004, Abcam, USA), and CD206 (1:500, ab64693, Abcam, USA)), and inflammatory and anti-inflammatory cytokines (TNF-α (1:500, ab1793, Abcam, USA), IL-1β (1:500, ab9722, Abcam, USA), IL-6 (1:500, ab9324, Abcam, USA), and IL-10 (1:250, ab9969, Abcam, USA)). The following morning, the sections were incubated with an HRP-conjugated secondary antibody for 2 h. Positive cells were then detected using DAB reagent.

### Image quantification and analysis

Images were analyzed by ImageJ. We set a threshold for each staining set in order to measure the number of cells of interest in immunohistochemical images. Only cells showing a signal above the set threshold were defined as positive cells. To determine the number of axons per group, we used the toluidine blue staining images to count all axons with a typical axonal structure. Using TEM images, we also determined the axonal area and the thickness of the myelin sheath. When determining the axonal area on the TEM images, we manually restricted the region of interest. When quantifying the thickness of the myelin sheath in each myelinated axon, we subtracted the diameter of the axon from the total diameter of the myelinated nerve fiber. Finally, pixel numbers were converted into actual surface area or actual length for final calculations.

### Statistical analysis

All data were analyzed by Origin (Pro), Version 8 (OriginLab Corporation, USA). One-way analysis of variance (ANOVA) and Fisher’s least significant difference (LSD) test were used to make comparisons between groups for each single condition. A *p* value < 0.05 was considered to indicate statistical significance.

## Results

### TM inhibited M1 inflammatory cytokine production and caused a switch toward the M2 phenotype

The combination of LPS and IFN-γ induction led to an M1 macrophage phenotype; thus, cells produced high levels of prototypical M1 cytokines, including *TNF-α*, *IL-1β*, and *IL-6* (Fig. 1b). Cells were harvested 24 h after TM incubation. The qPCR results showed that the administration of TM led to a significant decrease in the expression of *TNF-α*, *IL-1β*, *IL-6*, and *CD86* and a concomitant increase in the expression of genes encoding the M2 macrophage markers *IL-10* and *CD206* (Fig. 1c). These results indicate that TM promoted M2 polarization within an inflammatory microenvironment.

To gain further insight into the involvement of different domains of TM in the M1 to M2 transition, we incubated M1 macrophages with TM domain 1 (TMD1) or TM domains 2 and 3 (TMD23). The qPCR results showed that the administration of TMD1 led to a significant decrease in the expression of *TNF-α*. However, TMD1 could not augment the M2 markers *IL-10* and *CD206* (Supplementary Figure 1). In contrast, the administration of TMD23 not only reduced the expression of *TNF-α* and *CD86* but also increased *IL-10* and *CD206* levels (Supplementary Figure 1). These results reveal that TMD1 is involved in the anti-inflammatory function of TM and that TMD23 is required for the M1 to M2 transition.

**Fig. 2:**
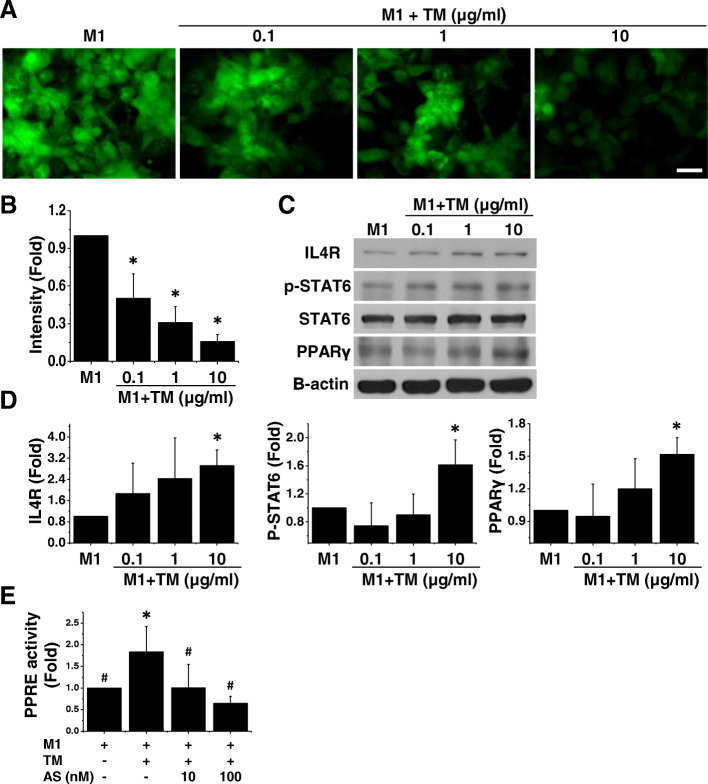
TM enhances M2 polarization by activating the STAT6-PPARγ pathway and regulating downstream reactive oxygen species (ROS) levels. **a** The ROS level was tested by using a ROS kit. Representative confocal images demonstrated that the levels of ROS were high in M1 macrophages. In contrast, TM-treated M1 macrophages showed low levels of ROS, irrespective of the dose of TM administered. Scale bar, 50 μm. *n* = 3. **b** Quantification of ROS level. **c** Western blotting data revealed a marked increase in IL-4R and phosphorylated STAT6 and a concomitant increase in the production of PPARγ. *n* = 3. **d** Quantification of Western blotting data. **e** PPARγ response element (PPRE) activity was measured by using a luciferase reporter assay. Reporter assay data revealed a marked increase in the activity of PPRE in TM-treated M1 cells. However, supplementation with STAT6 inhibitor (AS1517499, AS) caused a lower level of PPRE activity. *n* = 3. Mean ± SD. *p < 0.05 compared with M1. #*p* < 0.05 compared with TM-treated M1

TM is required for mitochondrial function and may therefore regulate the homeostasis of ROS levels. Intracellular mitochondrial ROS enhances macrophage polarization toward M1 [31]; therefore, we measured the levels of ROS in both M1 and TM-treated M1 cells. M1 induction led to an increase in ROS levels. In contrast, TM-treated cells exhibited a marked reduction in ROS production (Fig. 2a, b).

The IL-4 receptor (IL-4R) and its downstream effectors, including STAT6 and PPARγ, may be involved in oxidative metabolism and M2 macrophage polarization [32, 33]. To gain insight into the mechanism of action of TM in the IL-4R-regulated signaling pathway, we measured IL-4R expression in TM-treated M1 macrophages. Western blotting data revealed that TM-treated M1 cells exhibited a marked increase in IL-4R (Fig. 2c, d). In addition, we measured the phosphorylation of STAT6 and its downstream effector PPARγ. We observed a dose-dependent increase in the expression of pSTAT6 in M1 macrophages following the addition of TM. Concurrently, the production of PPARγ was increased in M1 cells treated with 10 μg/ml TM (Fig. 2c, d). To confirm the involvement of the pSTAT6-PPARγ signaling pathway, we blocked pSTAT6 with a STAT6 inhibitor (AS1517499) and used a PPARγ antagonist (GW9662) to bind the PPARγ ligand binding site. Both AS1517499 and GW9662 abolished TM-triggered *IL-10* and *CD206* levels (Supplementary Figure 2A). The promotor activity of PPRE in both M1 and TM-treated M1 macrophages was additionally tested by using a luciferase reporter assay. We observed a significant increase in the activity of PPRE following TM treatment, and inhibition of pSTAT6 significantly reduced that in TM-treated M1 cells (Fig. 2e). The c-Myc is a potent transcription factor involved in IL-4R-regulated M2 polarization and can upregulate STAT6 and PPARγ [32]; therefore, we incubated TM-treated M1 cells with a c-Myc inhibitor and found that c-Myc inhibition led to a significant decrease in the *CD206* expression level (Supplementary Figure 2C). These data further suggest that TM can activate the IL-4R-c-Myc-pSTAT6-PPARγ signaling pathway to switch M1 inflammatory macrophages toward the M2 phenotype.

### TM-conditioned media reduced SC inflammation

**Fig. 3:**
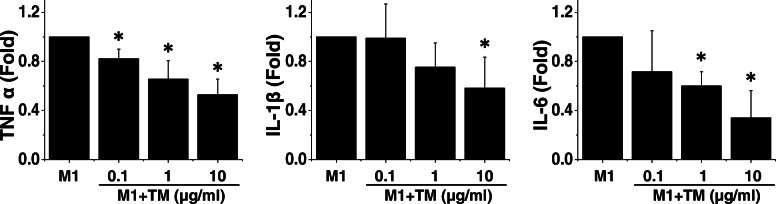
TM suppresses the ability of M1 macrophages to induce inflammation. Primary Schwann cells were incubated with conditioned medium derived from M0, M1, or TM-treated M1 cells. The expression level of inflammatory markers was measured by using quantitative RT-PCR. Quantitative RT-PCR data revealed that exposure to M1-conditioned medium triggered an increase in the RNA expression levels of *TNF-α*, *IL-1β*, and *IL-6* in Schwann cells, indicating the proinflammatory characteristics of factors secreted by M1 macrophages. The pro-inflammatory feature of M1 macrophages decreased following incubation with TM in a dose-dependent manner. *n* = 5. Mean ± SD. *p < 0.05 compared with M1

The potential effect of TM on PNS inflammation was tested using an in vitro primary SC model. Because SCs are the cornerstone of PNS regeneration, we incubated SCs with conditioned media derived from M0, M1, or TM-treated M1 cells to assess the secretory effect of these cells on SCs (Fig. 3). After 24 h of incubation, we determined the levels of *TNF-α*, *IL-1β*, and *IL-6* RNA in SCs. We found that when treated with M1-conditioned media, SCs produced inflammatory RNAs, thus indicating an inflammatory response. Conditioned media derived from TM-treated M1 cells exhibited a mild proinflammatory induction capability compared with that of M1 macrophages, thus indicating that TM can attenuate the inflammatory secretory factors derived from M1 cells in a dose-dependent manner (Fig. 3). Strikingly, the reduced proinflammatory capability of M1 cells treated with 10 μg/ml TM was comparable to that of M2 cells (data not shown). These data show that TM may prime macrophages into the functional M2 phenotype, even in the presence of inflammatory cytokines.

### The administration of TM promoted peripheral nerve regeneration and restored innervated muscle

**Fig. 4:**
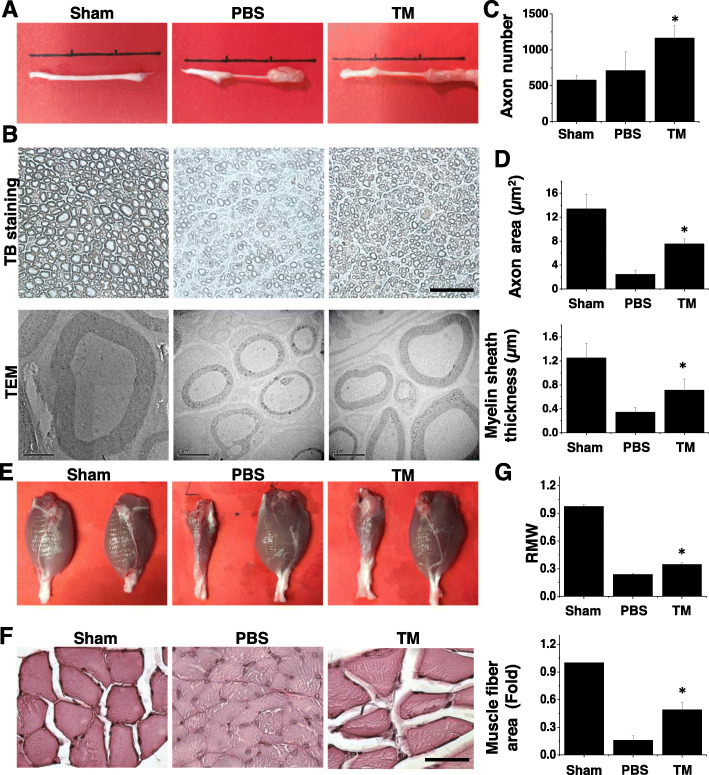
TM improves nerve regeneration after transverse nerve injury. **a** Gross view of regenerating sciatic nerves showing that TM-treated nerves have a wider diameter than those treated with PBS. **b** Light microscopic (upper row) and transmission electron micrographs (lower row) of sciatic nerve sections demonstrating an apparent increase in axon number, axon area, and myelin thickness in TM-treated nerve sections in comparison with those treated with PBS. Muscle mass and muscular structure were better preserved in the TM group. *n* = 3. **c** Quantification of axon number per unit, **d** axon area, and myelin sheath thickness. **e** Gross view of the gastrocnemius muscles. **f** Hematoxylin and eosin staining of gastrocnemius muscle sections showing an increase in RMW and muscle fiber in TM-treated nerves. *n* = 3. **g** Quantification of RMW and muscle fiber area. Scale bar, 100 μm (light microscopy). Scale bar, 2 μm (electron micrograph). Mean ± SD. *p < 0.05 compared with the PBS group

To investigate the therapeutic outcomes of TM treatment, we further examined the modulation of inflammation by administering TM to the site of peripheral nerve injury in a rat model. A TM concentration of 10 μg/ml led to the highest M1/M2 transition in our cell experiments; therefore, we used the same dose for animal experiments. The sciatic nerves were harvested 6 weeks after surgery (Fig. 4a). To assess nerve tissue regrowth, we carried out toluidine blue staining and transmission electron microscopy to observe the fine structure of the nerve (Fig. 4b). To gain a comprehensive understanding of nerve regeneration, we quantified three parameters: axon density, axon area, and myelin sheath thickness. We found that the application of TM significantly improved axon density, showing that TM could promote axon elongation either directly or indirectly (Fig. 4c). The TM-treated regenerating nerves also had a higher axon area and a higher myelin sheath thickness, indicating that TM might influence remyelination either directly or indirectly (Fig. 4d).

By investigating the weight of the gastrocnemius muscle, we can assess the extent of innervation between the nerves and the target muscle, which is dominated by the sciatic nerve. Even on gross examination, it was evident that there was a significant difference in the status of the gastrocnemius muscle between the PBS and TM groups (Fig. 4e). The muscle size in the TM group was larger than that of the PBS controls. To compare quantitative data, we compared the gastrocnemius muscles from the left side of the body with those from the right side and defined the ratio of the left muscle to the right muscle as the RMW. The RMW of the TM group was significantly higher than that of the PBS group (Fig. 4f). Correspondingly, muscle bundles were thicker and more intact in the TM group than in the PBS control group (Fig. 4g). These results indicate that the administration of TM promoted the retention of muscle mass and structure.

### TM modulated the tissue cytokine profile to create a proregenerative microenvironment

**Fig. 5:**
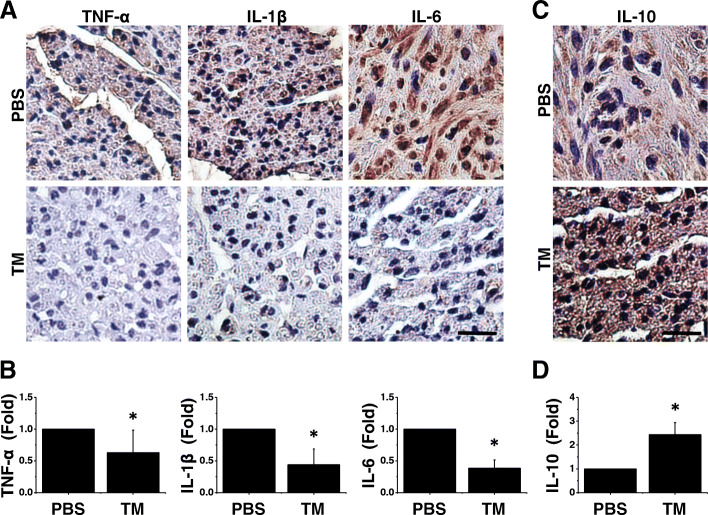
TM ameliorated the proinflammatory features of injured nerves. **a** Immunohistochemical staining of transverse nerve sections from Sprague-Dawley rats. Nerve sections from TM-treated nerves demonstrated an apparent reduction in IL-6 expression in comparison with that of nerves treated with PBS. *n* = 5. **b** Quantification of TNF-α, IL-1β, and IL-6 levels. **c** A concomitant increase in IL-10 was detected in nerve sections from the TM group. *n* = 5. **d** Quantification of IL-10 level. Scale bar, 20 μm. Mean ± SD. *p < 0.05 compared with the PBS group

Our earlier cell experiments indicated a reduction in the proinflammatory capability of M1 cells following incubation with TM. To clarify whether TM exerts an anti-inflammatory effect at the site of injury, we investigated the expression of proinflammatory and anti-inflammatory cytokines. In particular, we investigated the expression and localization of inflammatory cytokines, including TNF-α, IL-1β, and IL-6, to assess the microenvironment at the site of injury. Immunostaining revealed that the TM-treated nerves exhibited a marked decrease in TNF-α, IL-1β, and IL-6 expression (Fig. 5a, b). Correspondingly, the levels of IL-10 were higher in the TM group than in the PBS group (Fig. 5c, d). IL-10 facilitates tissue repair; therefore, this result may indicate that TM creates a favorable microenvironment for nerve regeneration.

### TM reduced glial scarring and pan-macrophage number by increasing the M2/M1 cell ratio

**Fig. 6:**
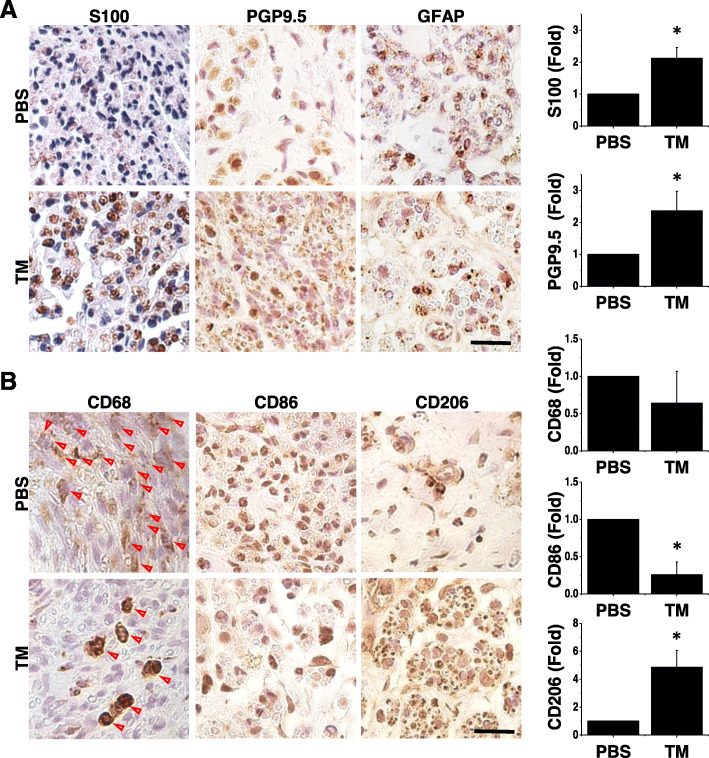
TM enhanced M2 polarization in a proinflammatory microenvironment. **a** Immunohistochemical staining of transverse nerve sections from Sprague-Dawley rats. S100 and PGP9.5 staining of a transverse nerve section demonstrated a higher proportion of Schwann cells and axons in TM-treated nerves. TM treatment also reduced the number of GFAP^+^ cells at the site of injury. *n* = 5. **b** Immunolabeling of CD68, CD86, and CD206 in nerve sections from the TM and PBS groups. Scale bar, 20 μm. *n* = 5. Mean ± SD. *p < 0.05 compared with the PBS group

To gain further insight into the beneficial effect of TM on nerve regeneration, we quantified the number of SCs and axons using antibodies against S100B and PGP9.5. We found that nerves in both the PBS and TM groups showed a normal myelin and axon structure, despite a significant reduction in the number of SCs (*p* < 0.05) and axon density (*p* < 0.05) in the PBS controls (Fig. 6a). In addition, we found only a small number of GFAP^+^ cells in the nerves treated with TM; in contrast, there was a large number of GFAP^+^ cells in the PBS controls. Because GFAP is a glial cell marker, a high concentration of GFAP^+^ cells at the site of injury is an indicator of glial scar formation. The small number of GFAP^+^ cells in TM-treated nerves showed that TM was able to suppress the formation of glial scars.

Given that TM is a key inflammatory modulator [34], we next aimed to investigate whether TM facilitates nerve regeneration via immune modulation. Our in vitro work showed that TM is able to induce the switch from M1 to M2. Therefore, we focused on the shift in macrophage phenotype following the application of TM. CD68 staining showed that while TM-treated nerves contained a lower number of infiltrated macrophages, there was no significant difference compared with the PBS group (Fig. 6b). To further define the subpopulation of these macrophages in the regenerating nerves, we quantified the number of M1 and M2 macrophages by staining tissue sections with antibodies against CD86 and CD206, respectively (Fig. 6b). Immunostaining showed that the macrophages within the vehicle group were mainly M1 macrophages, indicating that inflammation persisted in the nerves from the PBS group. In contrast, macrophages in the TM-treated nerves predominantly expressed the M2 marker, CD206. Our in vivo findings corresponded well with our in vitro studies involving THP-1 cells and provided compelling evidence for the role of TM in M1/M2 switching following nerve transection injury. Collectively, our data prove that TM favors the switching of macrophages to the M2 phenotype. We also demonstrated the potential of using TM as an immunoregulator to modulate inflammatory status and enhance nerve regeneration.

## Discussion

The dynamic balance between M1/M2 switching is critical during tissue repair. In the current study, we demonstrate the potential of TM in the modulation of macrophage polarization and show that TM can enhance nerve regeneration. Macrophage polarization can be triggered by a range of molecular signaling pathways. In this study, we demonstrate that TM promotes M1/M2 switching through the IL-4R-c-Myc-pSTAT6-PPARγ axis. STAT6 is the main transcription factor involved in IL-4R type 1-driven M2 polarization [35]. In addition, STAT6 is required for IL-4-induced PPARγ augmentation [36]. Although IL-4R type II can also trigger STAT6 activation, the production of *PPARγ* mRNA by macrophages is impeded only when Janus tyrosine kinase 3 (*JAK3*) is inhibited, and not *JAK2* [36]*.* This suggests that the TM-initiated STAT6-PPARγ pathway involves IL-4R type I, and not type II. In light of the wide-ranging anti-inflammatory capability that TM appears to possess, we believe that other potential pathways are involved in TM-induced M2 polarization, particularly in in vivo studies where complex cell-cell interactions exist.

In a previous study, Abeyama et al. demonstrated the ability of TM to sequester high mobility group B1 (HMGB1) through its lectin-like domain [37]. HMGB1 is secreted by necrotic cells and can enhance M1 polarization via TLR4 [38]. Therefore, HMGB1 is known to augment inflammation. Accumulating evidence shows that both neurons and SCs release HMGB1 following injury or stimulation by toxins [39]. Congruently, our in vitro study elucidated the ability of the lectin-like domain (TMD1) to suppress the expression level of *TNF-α*. We also observed a lower level of HMGB1 in TM-treated M1 macrophages (Supplementary Figure 3). These findings indicate that the sequestration of HMGB1 by the lectin-like domain represents another potential mechanism underlying TM-augmented M2 polarization in transected nerves. Furthermore, the EGF-like domain of TM can also exert anti-inflammatory effects by activating PC [7, 8]. However, it has been reported that recombinant TM fails to augment APC in rodents [40]. Current evidence appears to indicate that the mechanism underlying our current observations is not likely to involve APC-dependent immunoregulation.

In addition to ameliorating the inflammatory response, we also found that the application of TM can prevent the formation of glial scars. We believe that this therapeutic effect is caused by a lower level of HMGB1; previous studies have shown that elevated levels of HMGB1 are highly correlated with glial cell activation [41]. Previous literature also reports that the neutralization of HMGB1 in the diabetic spinal cord and transcended olfactory nerve with a neutralizing antibody reduces the production of GFAP [42, 43]. TM can sequester HMGB1 through its lectin-like domain; therefore, we consider that this mechanism might be responsible for the lower levels of GFAP in TM-treated nerves.

Although much of the existing literature supports the anti-inflammatory properties of TM, there is also evidence that TM regulates the immune system in other ways. For example, Wang et al. induced the differentiation of peritoneal macrophages in vivo by injecting thioglycollate and isolated cells from both wild-type mice and specific myeloid TM-null mutants [44]. These authors also showed that TM-deficient macrophages exhibited a significant reduction in the mRNA levels of several inflammatory cytokines, including *TNF-α*, *IL-6*, and *MCP-1*, as well as a lower level of ROS in comparison with that in wild-type macrophages, thus indicating the participation of endogenous TM in the induction of proinflammatory M1 cells [44]. Other studies have reported that both endogenous and soluble TM may regulate monocyte differentiation, as indicated by the increased expression of TM within monocytes during PMA induction [45, 46]. These findings indicate that TM may play diverse roles in regulating monocytes or macrophages depending upon the status of these cells. In the current study, we found that soluble TM can modulate the polarization of differentiated macrophages in transected nerves. However, the dynamic effect of TM on monocytes or macrophages in damaged nerves at different time points post-injury and how this affects nerve repair remain to be elucidated. Because acute inflammation and chronic inflammation appear to have different biological roles in nerve repair, TM may affect the immune system in a unique way during each phase of inflammation. The current study investigated the effect of TM during the late stages of inflammation. Therefore, future studies should place more emphasis on the effect of TM during the acute stage of inflammation in injured nerves.

Although M1 macrophages are reported to be proinflammatory, they are also indispensable for the nerve repair process [18]. This highlights the essential nature of an appropriate balance between the numbers of M1 and M2 macrophages to achieve better nerve regeneration. The current findings show that although TM enhances the transition of M1 to M2, some M1 macrophages were still found to persist in TM-treated nerves, indicating that TM may contribute to the maintenance of an ideal ratio of M1 to M2 instead of excessively suppressing the induction of M1. M2 macrophages perform multiple functions that are beneficial to nerve repair, including the suppression of inflammation and enhancement of angiogenesis [14, 47]. However, accumulating evidence also shows that macrophages are capable of performing many other functional roles. For example, previous literature has shown that macrophages promote the proliferation of SCs [48]. Macrophages are also involved in SC differentiation and remyelination via secreted ligands, such as Growth Factor Arrest 6 [16]. In the present study, we found that the number of SCs in the TM group was higher than that in the PBS control group. In addition to an increase in SC number, we also observed better remyelination in TM-treated nerves. Furthermore, macrophages are known to regulate SC migration and axon pathfinding in nerve bridges via Slit3-Roundabout Guidance Receptor 1 (Robo1) signaling [49]. Congruently, TM-treated nerves showed an increase in axon density. M1 and M2 macrophages perform unique functions and are both required for tissue repair. Collectively, our findings indicate that the application of TM enhances M2 polarization without interfering with the beneficial effect of macrophages in nerve repair. Moreover, the enhanced levels of M2 polarization boost nerve regeneration owing to the distinct proregenerative properties of M2 macrophages.

## Conclusions

In conclusion, our findings provide evidence that the application of TM can improve nerve repair by promoting M2 polarization. TM-treated M1 macrophages showed a reduction in M1 markers, including *TNFα*, *IL-1β*, *IL-6*, and *CD86*. In contrast, the expression of M2 markers, including *IL-10* and *CD206*, was upregulated following the application of TM. Our in vitro findings revealed IL-4R-c-Myc-pSTAT6-PPARγ to be the mechanism underlying TM-augmented M2 polarization. We also found that TM treatment led to increased numbers of SCs, increased axon density, better remyelination, and enhanced functional recovery.

## Supplementary information

**Additional file 1: Supplementary Figure 1. **TM domain 1 (TMD1) suppresses inflammation, while TMD23 enhances M2 macrophage polarization in the presence of inflammatory cytokines. The expression levels of M1 and M2 markers were tested by using quantitative RT-PCR. The quantitative RT-PCR data demonstrated that the addition of TMD1 caused a marked reduction in *TNFα* production. In contrast, TMD23 disrupted M1 polarization and enhanced polarization toward the M2 phenotype. *n* = 3. Mean ± SD. *p < 0.05 compared with M1.

**Additional file 2: Supplementary Figure 2. **Inhibition of STAT6, PPARγ, and c-Myc disrupts the ability of TM to enhance M2 polarization. (A) TM-treated M1 cells were incubated with 0.1, 1, and 10 μM PPARγ antagonist (GW9662, GW). The M2 markers IL-10 and CD206 were tested by using quantitative RT-PCR. The quantitative RT-PCR data demonstrated that antagonizing PPARγ caused a marked decrease in *IL-10* and *CD206* expression. *n* = 4. (B) TM-treated M1 cells were incubated with 10 and 100 nM STAT6 inhibitor (AS1517499, AS). The quantitative RT-PCR data demonstrated that inhibition of STAT6 markedly disturbed the expression level of *IL-10* and *CD206*. *n* = 5. (C) Inhibiting c-Myc with 60 μM c-Myc inhibitor (c-Myc i) resulted in a significant reduction in CD206 level. *n* = 5. Mean ± SD. *p < 0.05 compared with M1. #*p* < 0.05 compared with TM-treated M1.

**Additional file 3: Supplementary Figure 3. **The addition of TM suppresses the production level of high mobility group B1 (HMGB1) in the presence of inflammatory cytokines. (A) The production level of HMGB1 in M1 and TM-treated M1 cells were tested by using western blot. Western blotting data revealed a decrease in HMGB1 production level. *n* = 3. (B) Quantification of Western blotting data. Mean ± SD.

## Data Availability

All data generated or analyzed during this study are included in this published article [and its supplementary information files].

## Abbreviations

PNS: Peripheral nervous system
CNS: Central nervous system
TNF-α: Tumor necrosis factor α
IL: Interleukin
TLR: Toll-like receptor
TM: Thrombomodulin
EGF: Endothelial growth factor
PC: Protein C
APC: Activated PC
ERK: Extracellular signal-regulated kinase
LPS: Lipopolysaccharide
SC: Schwann cell
IFN-γ: Interferon-γ
FBS: Fetal bovine serum
PMA: Phorbol 12-myristate 13-acetate
SD rat: Sprague-Dawley rat
CM: Conditional medium
RMW: Relative muscle weight
SDS-PAGE: Sodium dodecyl sulfate-polyacrylamide gel electrophoresis
NC: Nitrocellulose
STAT: Signal transducer and activator of transcription
PPARγ: Peroxisome proliferator-activated receptor γ
HRP: Horseradish peroxidase
ROS: Reactive oxygen species
HMGB1: High mobility group B1
Robo1: Roundabout Guidance Receptor 1
