# Supplementary figures and images for: Thrombomodulin facilitates peripheral nerve regeneration through regulating M1/M2 switching
Source: J Neuroinflammation. 2020 Aug 21;17:240. doi: 10.1186/s12974-020-01897-z (PMC7477856; doi:10.1186/s12974-020-01897-z)

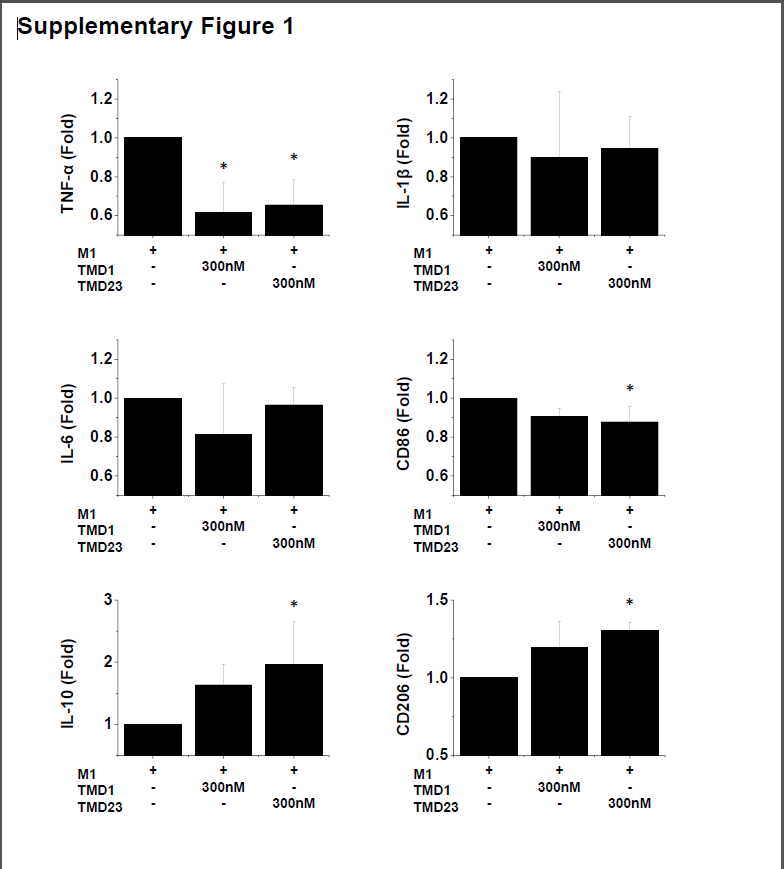

Supplement: Supplementary file 1 — Additional file 1: Supplementary Figure 1. TM domain 1 (TMD1) suppresses inflammation, while TMD23 enhances M2 macrophage polarization in the presence of inflammatory cytokines. The expression levels of M1 and M2 markers were tested by using quantitative RT-PCR. The quantitative RT-PCR data demonstrated that the addition of TMD1 caused a marked reduction in TNFα production. In contrast, TMD23 disrupted M1 polarization and enhanced polarization toward the M2 phenotype. n = 3. Mean ± SD. *p < 0.05 compared with M1. [file 12974_2020_1897_MOESM1_ESM.png]

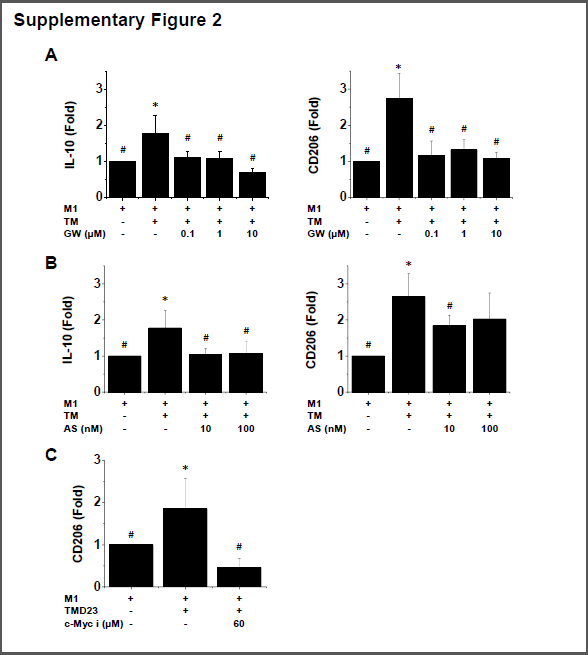

Supplement: Supplementary file 2 — Additional file 2: Supplementary Figure 2. Inhibition of STAT6, PPARγ, and c-Myc disrupts the ability of TM to enhance M2 polarization. (A) TM-treated M1 cells were incubated with 0.1, 1, and 10 μM PPARγ antagonist (GW9662, GW). The M2 markers IL-10 and CD206 were tested by using quantitative RT-PCR. The quantitative RT-PCR data demonstrated that antagonizing PPARγ caused a marked decrease in IL-10 and CD206 expression. n = 4. (B) TM-treated M1 cells were incubated with 10 and 100 nM STAT6 inhibitor (AS1517499, AS). The quantitative RT-PCR data demonstrated that inhibition of STAT6 markedly disturbed the expression level of IL-10 and CD206. n = 5. (C) Inhibiting c-Myc with 60 μM c-Myc inhibitor (c-Myc i) resulted in a significant reduction in CD206 level. n = 5. Mean ± SD. *p < 0.05 compared with M1. #p < 0.05 compared with TM-treated M1. [file 12974_2020_1897_MOESM2_ESM.png]

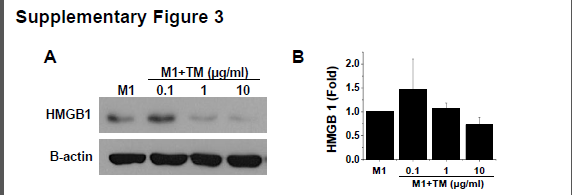

Supplement: Supplementary file 3 — Additional file 3: Supplementary Figure 3. The addition of TM suppresses the production level of high mobility group B1 (HMGB1) in the presence of inflammatory cytokines. (A) The production level of HMGB1 in M1 and TM-treated M1 cells were tested by using western blot. Western blotting data revealed a decrease in HMGB1 production level. n = 3. (B) Quantification of Western blotting data. Mean ± SD. [file 12974_2020_1897_MOESM3_ESM.png]
